# Evaluation of osteoblast response to polyacrylonitrile infused nano-curcumin coated on titanium discs: Invitro study cell culture experimental study

**DOI:** 10.1016/j.jobcr.2024.12.003

**Published:** 2024-12-09

**Authors:** Shilpi Gupta, N. Gopi Chander, Aravind Bhatt, K.V. Anitha

**Affiliations:** Department of Prosthodontics, SRM Dental College, Bharathi Salai, Ramapuram, Chennai, 89, India

**Keywords:** Curcumin, Titanium disc, Surface coating

## Abstract

**Purpose:**

The study evaluated the influence of titanium discs, coated with polyacrylonitrile infused curcumin nanofibers on osteoblast activity.

**Materials and methods:**

The titanium discs were coated with polyacrylonitrile nanofibers infused with curcumin. MG-63 cell lines were utilized for cell culture to assess osteoblast morphology upon exposure of curcumin on titanium discs. SEM comparison was made. Lactate Dehydrogenase (LDH) activity was measured after 2 and 7 days and the Alkaline Phosphatase (ALP) activity of the cells was quantified.

**Results:**

The results indicated that the coating had a notable impact on mineralization, LDH and ALP activities. Significant differences were observed between uncoated and coated samples. The SEM analysis indicated that curcumin enhanced bone growth when the Ti discs coated with curcumin are implanted in the bone.

**Conclusion:**

Polyacrylonitrile infused nano-curcumin fibers coated on titanium discs potentially enhanced osteoblast response and mineralization.

## Graphical abstract

**Figure:**
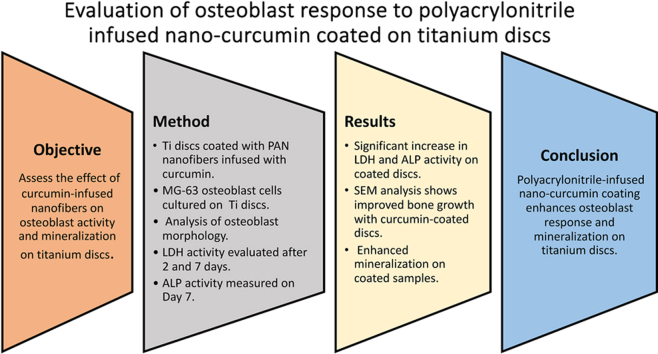

## 1 Introduction

Osseointegration is the union of dental implant and bone without any intervening fibrous tissue. Achieving osseointegration is imperative for ensuring the long-term success of dental implants. Numerous factors exert influence on the osseointegration of Titanium (Ti) dental implants within the host bone. Among the factors, the surface characteristics of the Ti implant play a pivotal role in determining the extent of osseointegration.^1^ The impact of microscopic features of the implant on cellular interactions and host responses has been recognized by several researchers. Surface modification attempts, including both additive and subtractive techniques have demonstrated enhanced osseointegration compared to unmodified machined Ti implants.^2^, ^3^, ^4^ Studies on sand blasted and acid etched (SLA) implants exhibited increased hydrophilicity and demonstrated the most favorable bone-implant contact when compared with other surface modified implants.^5^ Despite these advances, clinically implant failures are prevalent due to peri-implantitis, fibrous encapsulation or failed osseointegration.^6^

The clinical failures encountered in implant bioactivity have generated significant research interest in surface modification techniques aimed at enhancing their performance. Many proposed modifications have shown promise in encouraging fibroblast growth, although at the expense of inhibiting osteoblastic activity. Consequently, the pursuit continues for materials or techniques that can enhance osteoblastic activity effectively. Among the emerging strategies, the utilization of curcumin has garnered attention. Curcumin, recognized for its anti-inflammatory, antioxidant, and anti-carcinoma properties, has demonstrated potential in promoting bone regeneration and stimulating the osteogenic differentiation of osteoblasts.^7^ Markedly, few studies have observed that curcumin can effectively suppress fibroblast growth while concurrently stimulating osteoblasts around implants. However, curcumin, while promoting fibroblast activity in wound healing, has also shown potential in modulating fibroblast proliferation around implants, which can help prevent excessive fibrous tissue formation and promote osteoblast activity. Additionally, a significant concern arises on the effective bonding of curcumin to titanium surfaces, necessitating the use of a suitable binder to facilitate this process. Among the various potential binders explored, Polyacrylonitrile (PAN), a synthetic non-degradable polymer, has emerged as a promising material. PAN possesses desirable properties such as good biocompatibility, electrical conductivity, and antifungal characteristics ^8^^.^ The choice of utilizing curcumin as a bioactive agent and PAN as a binder is justified by the need to address the dual challenge of promoting osteoblastic activity while suppressing fibroblast growth around implants. This approach aids in enhancing the performance of implants and underscores the importance of innovative surface modification techniques in advancing biomedical applications. Fewer studies have evaluated PAN infused nano curcumin fibers on implants. Hence the study was done to determine the potential of Ti discs with PAN/curcumin nanofibers to enhance osseointegration and promote osteoblast response. Further exploration into the effects of different curcumin concentrations on osteoblast behavior holds the potential to optimize the titanium discs for enhanced osseointegration. Hence the study was aimed to determine the response of impact of PAN infused nano curcumin fibers on titanium discs by Invitro cell culture studies, Lactate dehydrogenase, alkaline phosphate activity to determine the cellular response, viability and osteoblast differentiation.

## 2 Materials and methods

The methodology employed for assessing the impact of curcumin-coated titanium discs on MG-63 cells was delineated into distinct subsections. The sample size of 30 titanium discs was determined by a power analysis using an alpha level of 0.05 and a power of 80 %, to detect significant differences in osteoblast activity.

### 2.1 Media and reagent preparation

The manufactured titanium (Ti) discs measuring 5 mm diameter × 3 mm height (SS Surgicals, Chennai) were used for the study. A range of media and reagents, including complete media, Dulbecco's modified Eagle medium (DMEM), PAN (molecular weight of 1,50,000),N,N-dimethylformamide (DMF) and curcumin were procured from Sigma-Aldrich for this study. Research. The preparation procedures adhered to standardized protocols. Subsequently, the media and reagents were sterilized using syringe filters and appropriately stored before utilization.

### 2.2 Electrospinning of PAN/curcumin nanofibers

The electrospinning parameters for PAN nanofibers were standardized by dissolving PAN solution in DMF. It was initially employed without the incorporation of curcumin. The solution was meticulously prepared by dissolving PAN in DMF at a concentration of 8 wt% with the aid of a magnetic stirrer. PAN nanofibers were subsequently produced using an electrospinner (E-Spinnano, Chennai) operating at a voltage of 20 KV, a collector-to-tip distance of 30 cm, and a flow rate of 0.2 ml/min. The morphology of the resulting nanofibers was analyzed using Scanning electron microscope (SEM).

Post standardization different weight percentages of curcumin were incorporated into the PAN/DMF solution to form distinct groups as outlined in Table 1. Nanofibers comprising PAN/curcumin were fabricated using identical parameters employed for PAN nanofibers, with SEM utilized for morphological analysis. The group exhibiting optimal morphology was subsequently selected for various in vitro studies, with results compared against those of control groups (non-coated Ti discs).

**Table 1:** Experimental groups with different wt% of curcumin.

Group	Wt% of curcumin
1	a
2	0
3	1
4	1.5
5	2

a Samples of group 1 comprised of non-coated Ti discs.

**Fig. 1:**
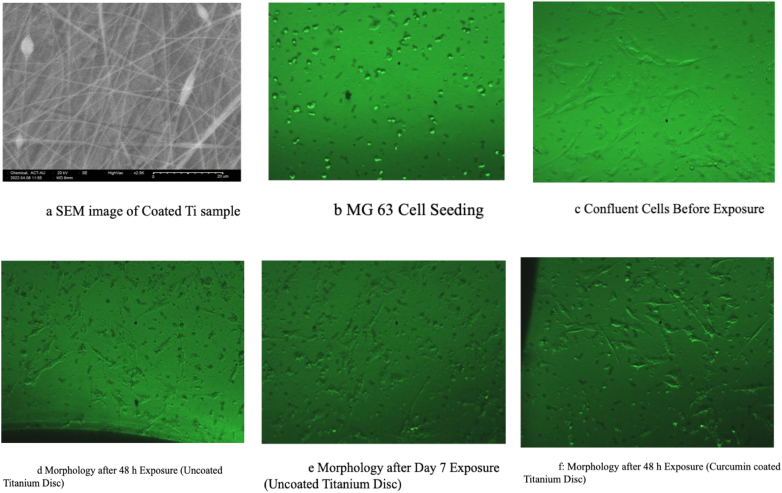
Morphology analysis images of titanium discs Fig. 1a: SEM image of Coated Ti sample Fig. 1b: MG 63 Cell Seeding Fig. 1c: Confluent Cells Before Exposure Fig. 1d: Morphology after 48 h Exposure (Uncoated Titanium Disc) Fig. 1e: Morphology after Day 7 Exposure (Uncoated Titanium Disc) Fig. 1f: Morphology after 48 h Exposure (Curcumin coated Titanium Disc).

The SEM image presented in Fig. 1 showcases the morphology of Ti discs coated with PAN/curcumin nanofibers across different groups. The SEM image elucidates the nanofiber morphology of the coated samples, revealing an increasing tendency toward beading with higher concentrations of curcumin. Among the various curcumin-containing nanofibers, group 3 exhibited the most uniform coating, thus warranting its selection for further investigations.

### 2.3 Cell line subculture

The MG-63 cell line (NCCS, Pune) and was subcultured in DMEM with NEAA media supplemented with 10 % FBS. Subculturing occurred when cell confluence reached 70–80 %. The procedure entailed discarding the media from the confluent flask and rinsing the cells thoroughly with calcium and magnesium-free phosphate-buffered saline (CMF-PBS). Subsequently, 0.25 % trypsin and EDTA were introduced to detach the cells. This detachment process was repeated two to three times to ensure complete detachment, followed by microscopic confirmation. Subcultured cells were utilized for further investigations.

### 2.4 Morphology assessment of MG-63 cells

MG-63 cells were seeded in a 96-well plate and incubated for 24 h at 37 °C with 5 % CO_2_ (Fig. 1a). After 24 h, the cells' morphology was examined (Fig. 1b). The MG-63 cell lines were then exposed to titanium discs, both with and without a curcumin coating, for 48 h at 37 °C with 5 % CO_2_ (Fig. 1c and e). The cellular morphology was subsequently assessed under a microscope on day 7 (Fig. 1d and f). The titanium discs were removed from the growth medium, fixed in a 4 % formaldehyde solution in phosphate-buffered saline (PBS), subjected to a series of alcohol dilutions, critically dried, and analyzed using SEM.

### 2.5 LDH activity determination

MG-63 cells were cultured in a 24-well plate and incubated at 37 °C with 5 % CO_2_ for 24 h. Curcumin-coated and uncoated titanium discs were exposed to MG-63 cell lines, with exposures lasting for 2 and 7 days at 37 °C with 5 % CO_2_. After the exposure period, cells associated with the titanium discs were observed under a microscope for morphology. Post-exposure, the LDH activity in the culture medium was measured. The culture media were collected, centrifuged at 500×*g* for 5 min at 4 °C, and the titanium discs were subsequently removed from the culture medium. The discs were fixed in a 4 % formaldehyde solution in PBS, subjected to alcohol dilutions, dried, and analyzed via SEM.

### 2.6 ALP activity determination

ALP activity was assessed by exposing MG-63 cells to curcumin-coated and uncoated titanium discs that were also used for LDH activity determination. Adherent cells were employed to determine ALP activity on day 7 of exposure. After washing adherent cells twice with cold PBS, they were detached by scraping in trypsin. The cells were then lysed in a 0.1 % Triton X-100 solution, centrifuged, and the resulting cell lysate was collected for ALP activity determination.

### 2.7 Mineralization assessment

**Fig. 2:**
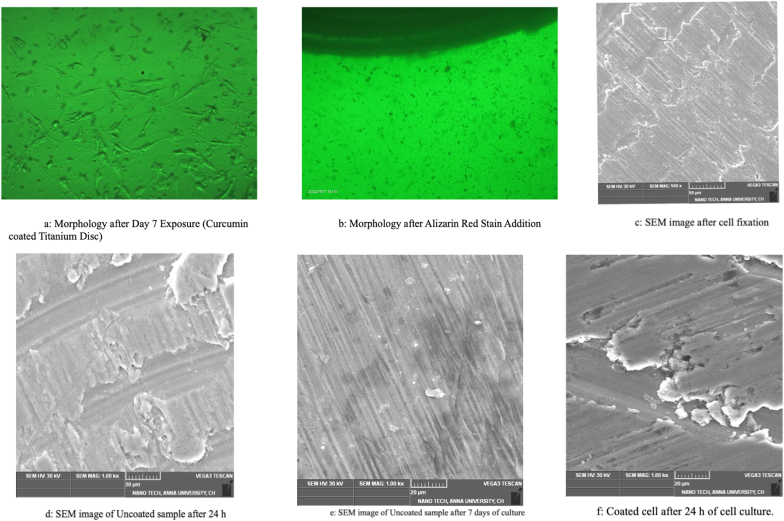
SEM and cell culture images of titanium discs Fig. 2a: Morphology after Day 7 Exposure (Curcumin coated Titanium Disc) Fig. 2b: Morphology after Alizarin Red Stain Addition Fig. 2c: SEM image after cell fixation Fig. 2d: SEM image of Uncoated sample after 24 h Fig. 2e: SEM image of Uncoated sample after 7 days of culture Fig. 2f: Coated cell after 24 h of cell culture. (For interpretation of the references to colour in this figure legend, the reader is referred to the Web version of this article.)

MG-63 cells were seeded at a density of 5 × 104 cells/well into a 96-well plate and incubated at 37 °C with 5 % CO_2_ for 24 h. After 24 h, the cells were examined for morphology. Curcumin-coated and uncoated titanium discs were sterilized via UV exposure for 15 min. Subsequently, the discs were exposed to MG-63 cell lines for 48 h at 37 °C with 5 % CO_2_. On day 4, after 48 h of incubation, the cells were observed under a microscope for morphology. The culture medium was aspirated, and the cells were rinsed and fixed with 95 % (v/v) ethanol for 15 min at 4 °C. Following ethanol removal, the cells were stained with 2 % Alizarin Red S in distilled water (adjusted to pH 4.1–4.3) for 15 min at room temperature. After aspirating the Alizarin Red S solution, the cells were rinsed with distilled water and destained with 33 % glacial acetic acid solution (Fig. 2 a and 2b).

### 2.8 Observation criteria

The study employed observation and evaluation criteria divided into four categories: morphology assessment, LDH activity determination, ALP activity determination, and mineralization assessment. Morphological changes were assessed through microscopic examination. LDH activity was determined in the supernatant culture media using a commercial biochemical kit from Biosystems and a biochemical analyzer after 2 and 7 days of exposure. ALP activity was determined using a commercial biochemical kit (Biosystems) and a biochemical analyzer after 7 days of exposure. Mineralization assessment involved measuring the absorbance of the destained cell solution, Optical Density (OD) with a spectrophotometer at 415 nm.

## 3 Results

Table 2 presents descriptive statistics for various parameters. On Day 2, the mean LDH levels were 172 ± 2.13 U/L for uncoated samples and 147.5 ± 3.41 U/L for coated samples. On Day 7, uncoated samples exhibited significantly higher LDH levels (256.5 ± 5.05 U/L) compared to coated samples (147 ± 2.64 U/L). ALP levels on Day 7 were higher in the coated group (70.38 ± 1.99 U/L) than in the uncoated group (51.24 ± 1.07 U/L). Furthermore, mineralization was enhanced in coated samples (0.092 ± 0.001 OD_415nm_) compared to uncoated samples (0.034 ± 0.002 OD_415nm_).

**Table 2:** Descriptive statistics.

		N	Mean ± S.D.
LDH Day 2 (U/L)	Blank	1	148
	Uncoated	30	172 ± 2.13
	Coated	30	147.5 ± 3.41
LDH Day 7 (U/L)	Blank	1	155
	Uncoated	30	256.5 ± 5.05
	Coated	30	147 ± 2.64
ALP Day 7 (U/L)	Blank	1	45.47
	Uncoated	30	51.24 ± 1.07
	Coated	30	70.38 ± 1.99
Mineralization (OD_415nm_)	Uncoated	30	0.034 ± 0.002
	Coated	30	0.092 ± 0.001

Table 3 displays the results of independent sample t-tests. All comparisons between uncoated and coated samples across LDH Day 2, LDH Day 7, ALP Day 7, and Mineralization were highly significant (p < 0.001), indicating substantial differences between the two groups. These findings confirm the effectiveness of the coating treatment in altering the parameters under investigation.

**Table 3:** Comparison of mean values between uncoated and coated samples using Independent sample *t*-test.

	df	Mean Differ-ence	S_E_	95 % CI		t	Sig. (2-tailed)
				Upper	Lower		
LDH Day 2	48.68	24.5	0.735	23.02	25.98	33.35	<0.001^a^
LDH Day 7	43.74	109.5	1.04	107.4	111.6	105.26	<0.001^a^
ALP Day 7	44.46	19.14	0.41	19.97	18.31	46.46	<0.001^a^
Mineralization	44.56	0.058	5.15 × 10^−4^	0.059	0.057	112.44	<0.001^a^

a p < 0.001.

Table 4 presents the Pearson correlation coefficients for LDH variables. Notably, LDH Day 2 and LDH Day 7 in uncoated samples showed a significant positive correlation (r = 0.371, p = 0.043), while other correlations were generally weak and non-significant, suggesting a limited impact from the coating. Additionally, Table 5 reveal non-significant correlations for ALP and mineralization between coated and uncoated samples, indicating that the coating did not significantly influence these parameters.

**Table 4:** Estimation of Pearson Correlation Coefficient for LDH activity.

VARIABLE		LDH DAY 2 UNCOATED	LDH DAY 2 COATED	LDH DAY 7 UNCOATED	LDH DAY 7 COATED
LDH DAY 2 UNCOATED	Correlation	–			
	p-value	–			
LDH DAY 2 COATED	Correlation	0.133	–		
	p-value	0.485	–		
LDH DAY 7 UNCOATED	Correlation	0.371^a^	0.105	–	
	p-value	0.043	0.580	–	
LDH DAY 7 COATED	Correlation	−0.061	0.260	−0.062	–
	p-value	0.748	0.165	0.744	–

a p < 0.05.

**Table 5:** Estimation of Pearson Correlation Coefficient for ALP activity and mineralization.

	DAY 7 COATED	
	Correlation	*P*-Value
ALP DAY 7 UNCOATED	0.032	0.868
Mineralization Uncoated	0.083	0.663

The results indicate that the coating treatment has a notable impact on LDH and ALP levels, as well as mineralization, with significant differences observed between uncoated and coated samples. The correlation analysis further suggests that while LDH levels in uncoated samples are related on different days, the coating has a limited impact on correlation patterns. These findings provide valuable insights into the effects of the coating treatment on the parameters studied, which could have implications for future research and applications in related fields.

**Fig. 3:**
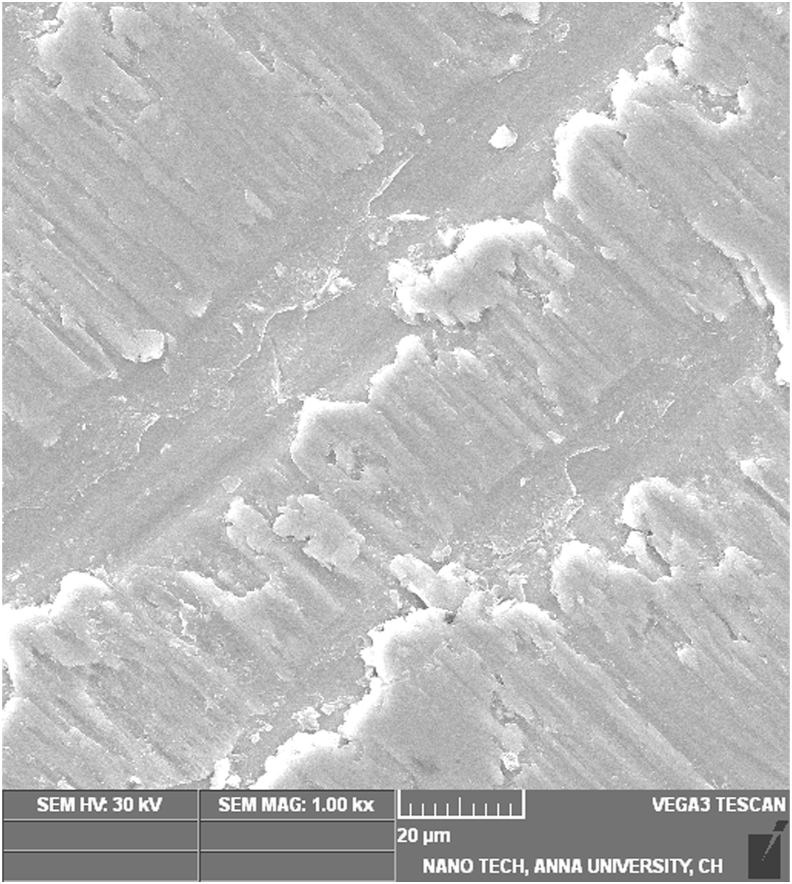
Curcumin coated Ti disc after 7 days of cell culture.

The SEM image of the Ti disc coated with PAN nanofibers containing curcumin displayed evidence of nanosize fibers (Fig. 2c). The beads observed in the fibers may be attributed to the presence of curcumin. The SEM image of the uncoated Ti sample, observed after cell fixation following 24 h of culture, suggests osteoblast cells adhering to the surface of the Ti disc, as well as surface mineralization on the sample (Fig. 2d). The analysis of the uncoated sample indicated several osteoblast cells adhering to the surface of the Ti disc, along with a thicker mineral layer compared to the 24-h sample. The curcumin-coated cells show a greater number of osteoblasts adhering to the surface of the Ti disc after 24 h compared to the uncoated sample after 24 h of cell culture (Fig. 2f). This distinctly suggests that curcumin favored a better osteoblast response. The SEM image of the sample after 7 days of cell culture demonstrates a large number of osteoblast cells attached to the surface, as well as a greater amount of mineral coating compared to the uncoated Ti discs after 7 days of cell culture (Fig. 2, Fig. 3). The SEM analysis indicates that curcumin enhances bone growth when the Ti discs coated with curcumin are implanted in the bone.

## 4 Discussion

In recent years, there has been a growing interest in leveraging surface modification techniques to enhance the osteointegration of titanium implants.^9^ The utilization of nanofibers composed of bioactive substances, such as curcumin, in conjunction with polymers like PAN, presents a promising avenue for achieving this goal. This discussion delves into the outcomes of a recent investigation aimed at assessing the influence of titanium implants coated with PAN/curcumin nanofibers on osteoblast response, with reference to pertinent literature.

The study's findings revealed no significant disparity in osteoblast morphology between cells exposed to curcumin-coated and untreated titanium discs. This observation suggests that the coating did not exert any adverse effects on cellular morphology. Consistent with prior research, curcumin has demonstrated its non-toxic and non-cytotoxic nature across various cell types, including osteoblasts.^10^^,^^11^

Conversely, the study identified an increase in LDH activity in cells exposed to uncoated titanium discs on Days 2 and 7 compared to those exposed to curcumin-coated discs. Elevated LDH activity typically indicates cellular damage. The reduced LDH activity observed in the presence of curcumin-coated titanium discs implies a protective effect against cellular harm or apoptosis. This protective effect aligns with previous investigations that showcased curcumin's capacity to mitigate cellular damage induced by oxidative stress in osteoblasts.^12^, ^13^, ^14^

ALP activity serves as an indicator of osteoblast differentiation and maturation. The study noted a modest elevation in ALP activity in cells exposed to curcumin-coated titanium discs, particularly on Day 7. This suggests that curcumin may enhance the differentiation and maturation of osteoblasts, corroborating prior research findings that attributed curcumin to improved osteogenic differentiation of mesenchymal stem cells and increased ALP activity in osteoblasts.^15^, ^16^, ^17^, ^18^, ^19^

Furthermore, the mineralization assay indicated a marginal increase in optical density (OD) at 415 nm for cells exposed to curcumin-coated titanium discs, implying a potential role of curcumin in encouraging mineralization in osteoblasts. This finding is in line with previous research demonstrating curcumin's ability to enhance calcium deposition and mineralization in osteoblasts.^20^^,^^21^

These results parallel existing studies that underscore the positive impact of curcumin on bone health. Beyond its potential to inhibit osteoclast activity and bone resorption, curcumin has been shown to promote osteoblast proliferation, development, and mineralization. Additionally, the anti-inflammatory and antioxidant properties of curcumin contribute to supporting bone healing and regeneration ^22^^,^^23^^.^

While this study provides valuable insights into the potential application of titanium implants coated with PAN/curcumin nanofibers for enhancing osteoblast response, several limitations must be addressed. Firstly, the study was conducted in vitro, limiting the generalizability of the findings to clinical settings. Moreover, the study predominantly explored short-term effects, necessitating further investigation to elucidate long-term impacts.

The study focused on titanium discs, extending the application of curcumin coatings to various implant materials is imperative. Future research should also explore the impact of curcumin-coated titanium implants on other cell types, such as osteoclasts and mesenchymal stem cells, which play pivotal roles in bone healing and regeneration. Expanding the sample size to encompass larger populations will enhance the robustness of the outcomes. Furthermore, future studies should consider in vivo models to validate the effects on osteoblast response and bone formation. Evaluations of the coating's efficacy and toxicity at varying curcumin and PAN concentrations are warranted. Additionally, investigations into the coating's ability to reduce bacterial adhesion and biofilm formation are crucial in the context of implant-associated infections.

The results of this study suggest that curcumin-coated titanium implants may hold promise in positively influencing osteoblast response and mineralization. This investigation serves as a promising starting point for further research into the utilization of PAN/curcumin nanofiber coatings, with potential clinical implications for improving implant outcomes and enhancing the quality of life for patients.

## 5 Conclusion

Curcumin-coated titanium implants show promise in enhancing osteoblast response and mineralization, necessitating further validation and exploration in diverse contexts.

## Patient consent statement

Not applicable.

## Ethical statement

No ethical conflicts.

## Permission to reproduce materials

Yes.

## Ethical clearance

Invitro study design. Not applicable for this study.

## Patient/guardian consent form

. Invitro study design. Not applicable for this study.

## Clinical trial registration

Not applicable.

## Funding

The study was supported as student grant by International team of Implantology- India. The recipient of the funds is the primary author of the study.

## Declaration of competing interest

The authors declare the following financial interests/personal relationships which may be considered as potential competing interests: Shilpi Gupta reports financial support was provided by 10.13039/501100016070ITI International Team for Implantology. If there are other authors, they declare that they have no known competing financial interests or personal relationships that could have appeared to influence the work reported in this paper.
